# Relationship between Activity in Human Primary Motor Cortex during Action Observation and the Mirror Neuron System

**DOI:** 10.1371/journal.pone.0004925

**Published:** 2009-03-17

**Authors:** James M. Kilner, Jennifer L. Marchant, Chris D. Frith

**Affiliations:** 1 The Wellcome Trust Centre for Neuroimaging, London, London, United Kingdom; 2 Niels Bohr Project “Interacting Minds”, CFIN, University of Aarhus, Århus, Denmark; James Cook University, Australia

## Abstract

The attenuation of the *beta* cortical oscillations during action observation has been interpreted as evidence of a mirror neuron system (MNS) in humans. Here we investigated the modulation of *beta* cortical oscillations with the viewpoint of an observed action. We asked subjects to observe videos of an actor making a variety of arm movements. We show that when subjects were observing arm movements there was a significant modulation of *beta* oscillations overlying left and right sensorimotor cortices. This pattern of attenuation was driven by the side of the screen on which the observed movement occurred and not by the hand that was observed moving. These results are discussed in terms of the firing patterns of mirror neurons in F5 which have been reported to have similar properties.

## Introduction

Mirror neurons were first discovered in premotor area F5 of the macaque monkey [1]–[4] and subsequently in rostral inferior parietal lobule, area PF [5]–[6]. Mirror neurons discharge not only when the monkey performs an action but also when the monkey observes a person performing the same action. A number of neuroimaging studies have claimed that a mirror neuron system (MNS) exists in humans and that homologous areas in the human brain are similarly activated when observing and executing movements [7]–[10], [Bibr pone.0004925-Morin1] [see references in 11 and 12 for a comprehensive list of previous neuroimaging studies]. However, over a decade after their discovery there is still debate as to whether any of the human neuroimaging studies constitute conclusive evidence for mirror neurons in humans [13].

Neuroimaging studies employing EEG or MEG have demonstrated an attenuation of cortical oscillatory activity during periods of movement observation that is similar to that observed during movement execution in both the 8–12 Hz (*mu*) range and the 15–30 Hz (*beta*) range [14]–[17]. The attenuation of the *beta* oscillations during action observation has been interpreted as evidence of a MNS in humans [18]. Although it is well established that this synchronous oscillatory activity in the *beta* range principally originates in primary motor cortex (M1) [19]–[21] it has been argued that, given the anatomical connection between inferior frontal gyrus and M1 [22]–[23], M1 is activated postsynaptically during periods of action observation.

Here we tested whether beta attenuation was modulated by the hand that was being observed or whether the beta attenuation was modulated by the side of the screen on which the movement occured. To this end we recorded cortical activity of human subjects using whole-head magnetoencephalography (MEG) whilst they watched a series of videos of an actor making arm movements. The results show that the pattern of beta attenuation was modulated by the viewpoint of the observed action and not the hand that was observed moving. These results are discussed in terms of the firing patterns of mirror neurons in F5.

## Results

### Modulations of *beta* power in sensor space: Experiment 1

**Figure 1:**
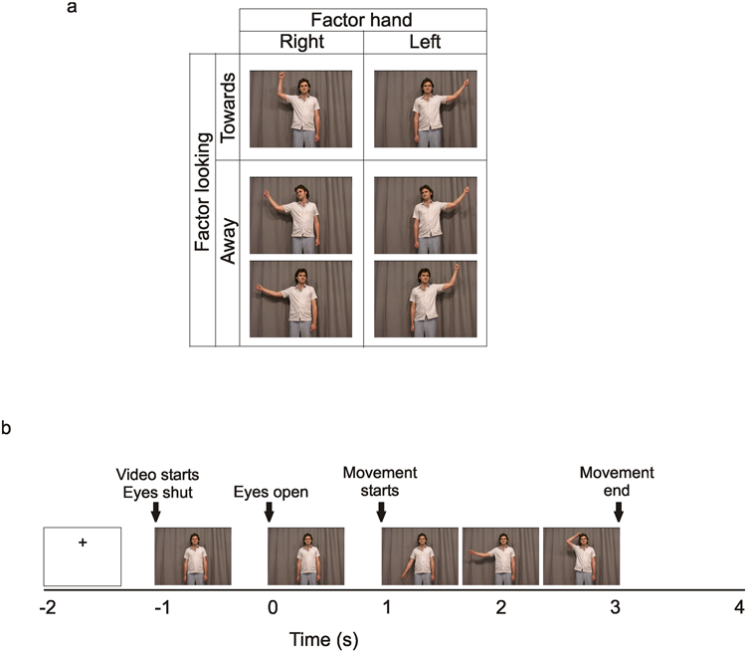
Experimental design. Figure 1a stills from the videos of experiment 1 showing the 2×2 factorial design collapsed across the factors goal and eye gaze direction. The factors depicted here are hand moved and the head position. Figure 1b shows the time course of a typical trial.

**Figure 2:**
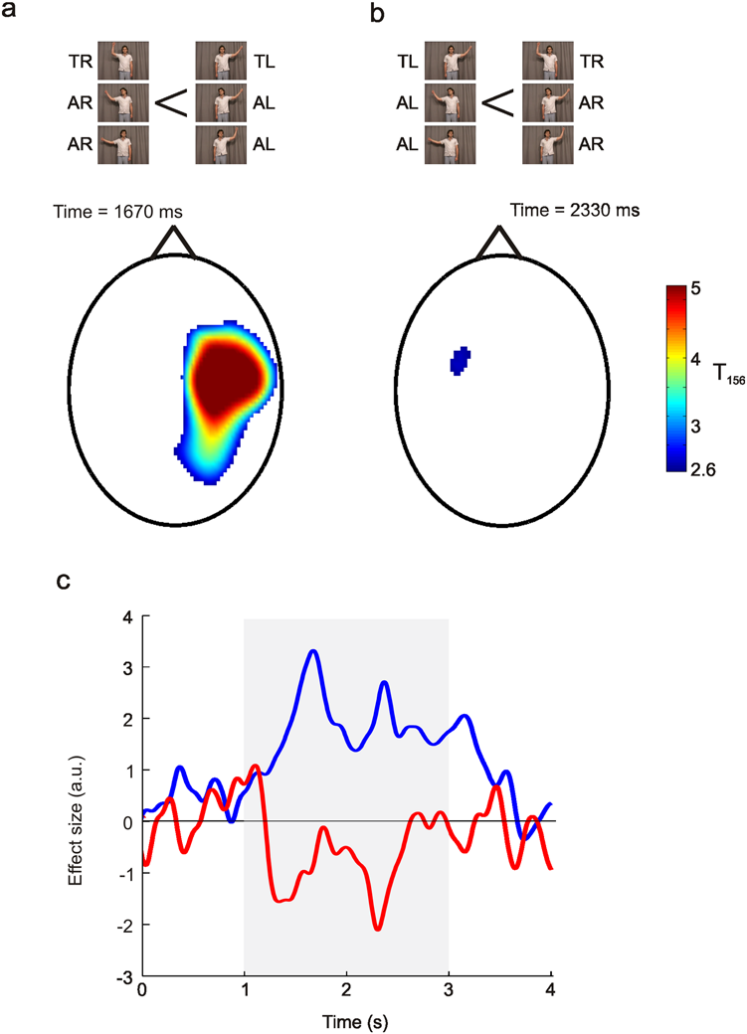
Sensor space statistical parametric maps. Figure 2a shows the statistical parametric map where *beta* power was smaller when observing a right handed action then when observing a left handed action in sensor space. Figure 2b shows the statistical parametric map for the interaction between the hand observed and the direction the actor's head was facing. The colour-scale in both (a) and (b) depicts the t-value. These statistical maps were thresholded at p<0.005 (uncorrected). Figure 2c shows the time course of the betas (a.u.) averaged across voxels that were above the p<0.005 threshold that are shown in panels (a) and (b). The blue line shows the time course for the beta value for the contrast shown in panel a The red line shows the time course for the beta value for the contrast shown in panel b. The grey box shows the period of action observation. TR – towards right hand, TL – towards left hand, AR – away right hand, AL – away left hand. It should be noted that the reciprocal nature of the modulations in the left and right hemisphere can not simply reflect extrema of classic dipolar field patterns as here we consider sensor space maps of power where the data has been squared and, therefore, is always positive.

Analysis of the 2×2 factorial design shown in Fig. 1A revealed two contrasts that showed significant modulations in *beta* power during the period of action observation. Firstly there was a main effect of the hand observed (Fig. 2A). *Beta* power was significantly more attenuated (peak voxel t = 6.21, p<0.05 corrected for multiple comparisons) at sensors overlying the right sensorimotor cortex when the subjects observed movements of the right hand compared to the left hand. This effect was observed throughout the 2 s period of action observation (blue line Figure 2C) and peaked at 1670 ms, 670 ms after movement onset. No voxels were significant for the reverse contrast (p>0.005). In other words there were no voxels where *beta* power was more attenuated when observing all movements of the left hand compared to the right hand. However, there was a significant interaction between the hand observed and the direction the actors' head was facing (Fig. 2B). This modest yet significant modulation of *beta* power (peak voxel t = 2.80, p<0.005 uncorrected) was observed at sensors overlying the left sensorimotor cortex and peaked at 2330 ms, 1330 ms after movement onset (red line Fig. 2C).

**Figure 3:**
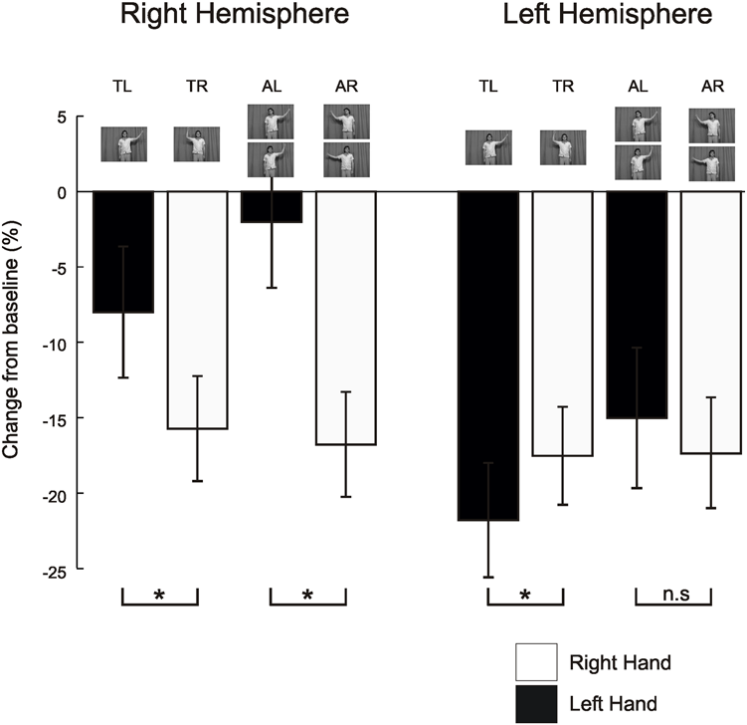
Responses at the peak voxel. Figure 3 shows the average *beta* power, represented as a percentage change from baseline, which was defined as the 1 s before video onset, from the taken from the peak voxels in Figures 2a and b. White boxes show averages when watching a right hand and black boxes show the averages when subjects were watching a left hand. The different conditions are depicted by the stills from the movies. Significant differences are shown with a * p<0.05. TR – towards right hand, TL – towards left hand, AR – away right hand, AL – away left hand.

Subsequent analyses of these effects focussed on the modulations of *beta* power at the peak voxel indentified from the two significant SPMs described above. These results are shown in Figure 3. The analysis of these data was in the form of a repeated measures 2×2×2 ANOVA where the factors were hemisphere (Right or Left), the direction the actors' head was facing (towards or away) and the observed hand that was moving (Left or Right). The results of this repeated measures 2×2×2 ANOVA revealed a main effect of hemisphere (F(1,12) = 12.7, p<0.05), a main effect of direction of the actors' head (F(1,12) = 6.0, p<0.05), a main effect of the hand observed moving (F(1,12) = 15.3, p<0.05), and a significant interaction between hemisphere and the hand observed moving (F(1,12) = 17.7, P<0.05). Post-hoc t-test revealed that for data recorded over the right hemisphere *beta* power was significantly more attenuated (p<0.05) when subjects observed a right hand than when they observed a left hand irrespective of the direction of head gaze (Fig. 3). Whereas for data recorded over the left hemisphere the converse was true. For the left hemisphere *beta* power was significantly more attenuated (p<0.05) when subjects observed a left hand compared to when they observed a right hand. However, this was only the case when the actor was facing forward; when the actor was facing away there was no significant modulation in the degree of *beta* power attenuation (p>0.3). It is important to note that one would expect an interaction between the hemisphere and the hand to be significant as the voxels of interest were selected based upon these contrasts. The purpose of reporting it here is to show that the same result is produced when using pooled or partition variance estimates. However, all other significant effects would not necessarily be predicted as they are orthogonal contrasts.

**Figure 4:**
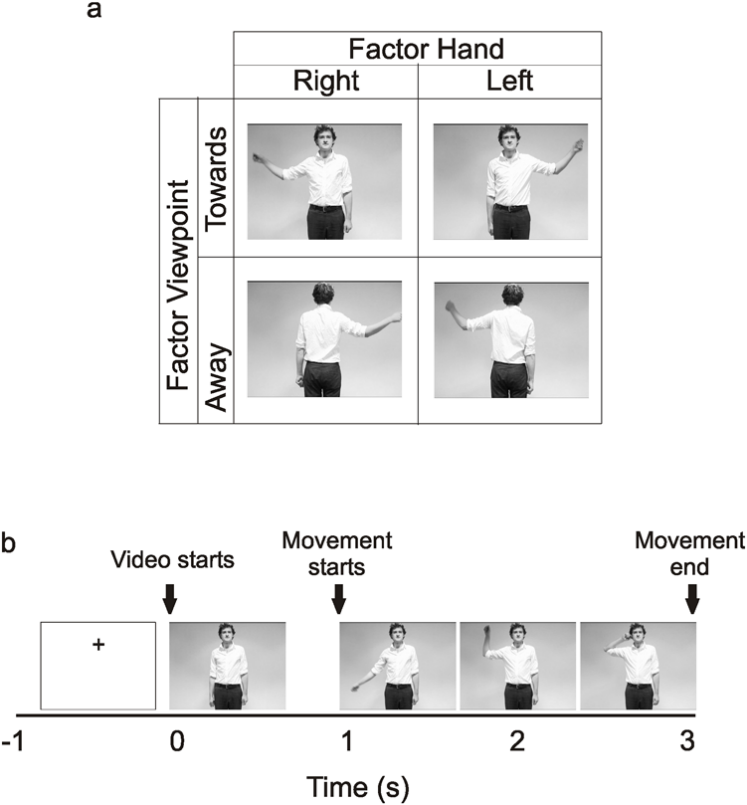
Experimental design. Figure 4a stills from the videos of experiment 2 showing the 2×2 factorial design collapsed across the factors goal. The factors depicted here are hand moved and the viewpoint. Figure 4b shows the time course of a typical trial.

These analyses revealed two things. First, *beta* power was more attenuated in the left hemisphere than the right hemisphere irrespective of the movement observed. Second, *beta* power was more attenuated in the right hemisphere when subjects observed a movement of the right hand and conversely, when the actor was facing forward, *beta* power was more greatly attenuated in the left hemisphere when subjects observed a movement of the left hand. However, the results of **experiment 1** are problematic to interpret as the hand observed moving was always on one side of the screen. In other words, a movement of the right hand always occurred on the left of the screen and a movement of the left hand always occurred on the right of the screen. In **experiment 2** this was controlled for as the movement of the left and right hands occurred on both the left and right of the screen (Fig. 4A). Analysis of the modulation of *beta* power in **experiment 2** would reveal whether the effects observed in **experiment 1** were being driven by the hand observed or by the side of the screen on which the movement occurred.

### Modulations of *beta* power in sensor space: Experiment 2

**Figure 5:**
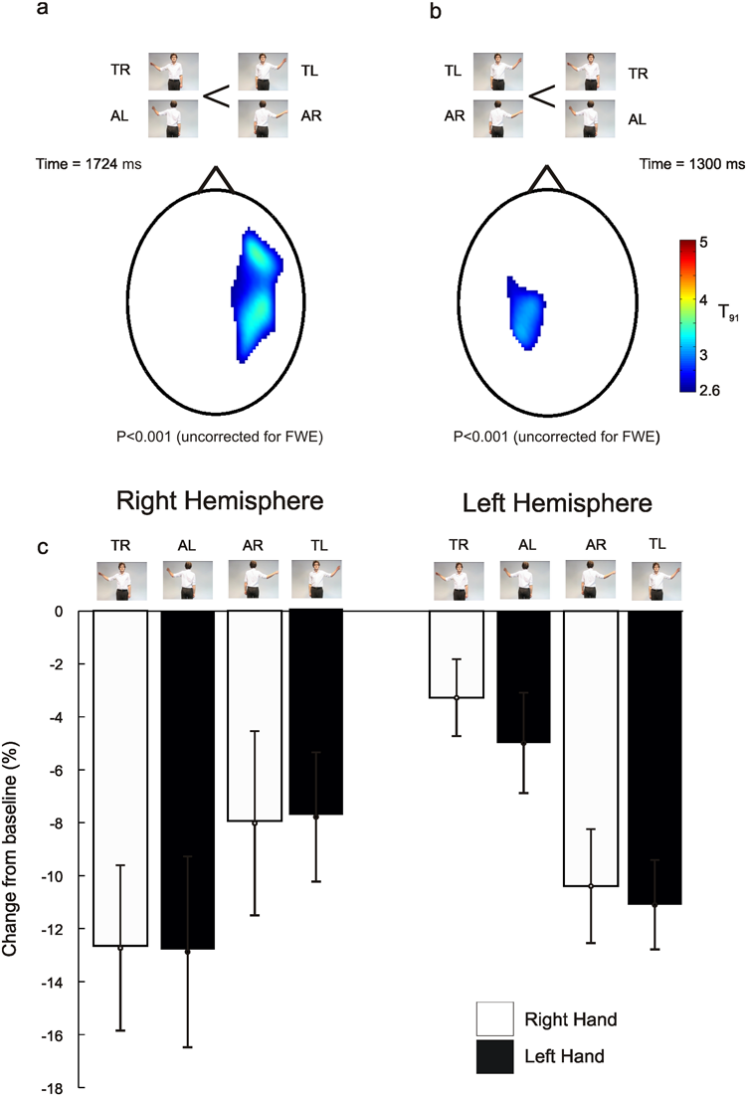
Sensor space statistical parametric maps. Figure 5a shows the statistical parametric map where *beta* power was smaller when observing a movement on the left of the screen than when observing a movement on the right of the screen. Figure 5b shows the opposite statistical parametric map where *beta* power was smaller when observing a movement on the right of the screen than when observing a movement on the left of the screen. The colour-scale in both (a) and (b) depicts the t-value. These statistical maps were thresholded at p<0.005 (uncorrected). Figure 5c shows the average *beta* power, represented as a percentage change from baseline, which was defined as the 1 s before video onset, from the taken from the peak voxels in panels a and b. White boxes show averages when watching a right hand and black boxes show the averages when subjects were watching a left hand. The different conditions are depicted by the stills from the movies. TR – towards right hand, TL – towards left hand, AR – away right hand, AL – away left hand.

Analysis of the 2×2 factorial design shown in Fig. 4A revealed that the only contrast that showed significant modulations in *beta* power during the period of action observation was the interaction between the hand observed and the direction the actor was facing (Fig. 5). In other words, the attenuation of *beta* power was modulated by the side of the screen on which the observed action occurred. *Beta* power was significantly more attenuated (peak voxel t = 3.90, p<0.001 uncorrected) at sensors overlying the right sensorimotor cortex when the subjects observed movements on the left of the screen compared to the right. This effect was maximal at 1724 ms, 724 ms after movement onset. For the reverse contrast *beta* power was significantly more attenuated (peak voxel t = 3.37, p<0.001 uncorrected) at sensors overlying the left sensorimotor cortex when the subjects observed movements on the right of the screen compared to the left. This effect was maximal at 1300 ms, 300 ms after movement onset. No voxel showed a significant main effect of the hand observed (p>0.005 uncorrected) during the period of action observation.

Subsequent analyses of these observed effects focussed on the modulations of *beta* power at the peak voxel indentified from the significant SPMs described above. These results are shown in Fig. 5B. The analysis of this data was in the form of a repeated measures 2×2×2 ANOVA where the factors were hemisphere (Right or Left), the direction the actor was facing (towards or away) and the observed hand that was moving (Left or Right). The results of this repeated measures 2×2×2 ANOVA revealed that only the interaction between the hemisphere, the direction and the hand was significant (F(1,13) = 23.8, p<0.05) all other main effects and interactions were not significant (p>0.2). As before, one would expect this interaction to be significant as the voxels of interest were selected based upon these contrasts. However, the purpose of reporting it here is to show that the same result is produced when using pooled or partition variance estimates.

## Discussion

The attenuation of the *beta* oscillations during action observation has been interpreted as evidence of a MNS in humans. In agreement with previous studies we show that during the period when subjects were observing the movements there was a significant attenuation of *beta* oscillations overlying left and right sensorimotor cortices. In **experiment** 1 we found that *beta* oscillations overlying the left and right hemispheres were differentially attenuated by the hand that was observed moving with the attenuation of *beta* oscillations during action observation greatest overlying the sensorimotor cortex ipsilateral to the hand that was observed moving. In **experiment 2** we found that this pattern of attenuation was driven by the side of the screen on which the observed movement occurred and not by the hand that was observed moving. *Beta* oscillations were more attenuated at sensors overlying the sensorimotor cortex contralateral to the side of the screen on which the observed movement occurred.

### Origin of the *beta* oscillations

There are several lines of evidence that suggest that the *beta* oscillatory activity at ∼20 Hz at sensors overlying sensorimotor cortex primarily originates in M1. First, invasive recordings of neurons within M1 of non-human primates have shown synchronous oscillatory activity at ∼20 Hz [19], [24]. Second, *beta* oscillatory activity in the 15–30 Hz range influences descending motor commands to contralateral hand muscles [19], [24]–[31]. Third, models of the location of the generator of the neuromagnetic *beta* activity recorded from humans find the most likely source in M1 [20], [27] although it should noted that a previous study has also found sources in other cortical areas as well as M1 [32].

### Relationship between M1 and the MNS

M1 is not considered part of the MNS. The MNS is often considered to consist of three reciprocally connected areas, ventral premotor area F5, inferior parietal lobule, area PF, and the superior temporal sulcus (STS; [33], [34]). Of these three areas mirror neurons have only been reported in two areas, F5 and area PF.

Despite not being considered part of the MNS it does appear beyond doubt that activity in the M1 is modulated during action observation. First, cortical oscillations that originate in the M1 are modulated when subjects are observing actions [17], [35]. Second, when M1 is stimulated using transcranial magnetic stimulation (TMS) the motor evoked potentials (MEPs) generated in contralateral hand muscles are augmented when subjects observe actions involving the hands compared to control conditions [36]–[38]. Finally, observed actions have been shown to have a measurable interference effect on simultaneously executed actions [39]. It has been argued that studies that investigate modulations of activity in M1 are indirectly studying activity in the MNS as a consequence of the strong reciprocal cortico-cortical connections between M1 and ventral premotor area, F5. The strength of these connections is such that activity in F5 can influence the corticospinal drive to hand muscles by modulating the sensitivity to stimulation of neurons in M1 [40].

### Relationship to previous studies

Here we have shown that attenuation of beta oscillations during action observation was modulated by the side of screen the action occurred and not the hand performing the action. This result is consistent with previous electromagnetic studies that have used a third person viewpoint of the observed action and reported similar modulations in the lateralised readiness potential [41], [42] and in the degree of augmentation in the beta oscillations after the observed action had ended [43].

The conclusion of the current study, that the M1 is more active contralateral to the side of the screen on which an observed movement occurs, is also consistent with the results of previous studies that have investigated the activity of M1 during action observation [37], [38], [44]. It is consistent with TMS studies that have reported that left and right primary motor cortices were more strongly activated when viewing actions conducted by the contralateral hand [37]. Furthermore, in the current study we replicate the observation by Aziz-Zadeh et al. [37] that the left M1 is active independent of the hand observed. It is consistent with an fMRI study that demonstrated that observation of a movement by a right hand (that appeared on the right side of the screen) resulted in activity in left M1 and conversely when the movement was performed by a left hand (that appeared on the left of the screen) activity was greatest in the right M1 [44]. The results of previous studies have been interpreted as evidence of a human MNS because the pattern of results observed during action observation matched those observed during action execution. The results of the current study suggest that this was only the case because of the nature of the stimuli used. In other words when an action is observed from a first person perspective the right hand is always on the right of the screen and the left hand is always on the left making it impossible to dissociate effects of hand from those of side of space the action ocurred. As far as we are aware there is only one study that has investigated the role of viewpoint on activity in the M1. This was a TMS study that stimulated the left M1 whilst subjects watched right handed actions that were presented both in an egocentric and allocentric perspective [38]. In agreement with the results presented here, they found that facilitation of the right hand, by stimulation of the left M1, was greater when subjects were watching the stimuli in the first person perspective compared to the third person perspective.

### Modulation of *beta* oscillations be social relevance

Previously we have reported that parietal alpha oscillations are modulated by the social relevance of the observed action [45].When subjects observed actions when the actor was facing the subject the alpha rhythm was augmented in the hemisphere ipsilateral to the side of visual space that the observed movement occurred. Such a pattern is consistent with the fact that subjects were attending to the side of the screen where the movement occurred. However, what was surprising was that this modulation did not occur when the actor was facing away. We argued that this difference might reflect a modulation in visuospatial attention by the social relevance of the actor whose actions were observed. In agreement with this, we have shown in **experiment 1**, that at sensors overlying the left hemisphere, the difference in beta power between observed left and right hand movements was only significant when the actor was looking at and turned towards the subject. When the subject was looking towards the corner of the room this effect was no longer significant (Fig. 3). However, such modulations were only observed when the actor was facing the subject and had their eyes or head averted. In the second experiment when the actor was facing away from the subject there was no modulation in the degree of *beta* attenuation compared to when the actor was facing forward (Fig. 5).

### Possible mechanisms underlying the modulation of beta attenuation during action observation

In this section we will speculate on possible neural mechanisms that could be consistent with the results observed. Previous studies have interpreted the attenuation of beta oscillations during periods of action observation as evidence of mirror neurons in the human motor system [14]–[17]. Mirror neurons in both left and right F5 of the macaque monkey respond to the observation of actions performed by both the right and the left hands [2]. Furthermore, these mirror neurons in F5 have been shown to be modulated by the side of the visual space in which the observed action occurred. To quote from Gallese et al. [2]:

“Nine neurons responded more strongly when the experimenter used the hand ipsilateral to the monkey's recorded hemisphere (i.e. the experimenter's left hand when the recorded hemisphere was the left one and the experimenter's right hand when the recorded hemisphere was the right one), … The preferred hand during active movements was, however, the right hand i.e. the hand opposite to that evoking the best visual responses. Note that in the case of face to face stance, the hand of an acting individual corresponds spatially to the opposite hand of the observing individual.”

This pattern of modulation is identical to the modulation of *beta* oscillations we have observed in the current study. Therefore, the results presented here are consistent with the hypothesis that, during periods of action observation, M1 is activated postsynaptically through anatomical connection between mirror neurons in inferior frontal gyrus and M1.

If we accept that mirror neuron activity is driving the effect we see here then the question remains as to what is modulating the response of mirror neurons in left and right inferior frontal gyrus when actions are viewed on the left and right of the screen? Here we will focus on three different possible mechanisms that explain behavioural findings when subjects are asked to imitate or respond to observed actions. These are a stimulus-response compatibility account (S-R compatibility effect), a goal-directed account, and a perspective taking account.

The pattern of responses reported here is consistent with a S-R compatibility effect. In its simplest form the S-R compatibility effect is the observation that, if a subject is asked to respond to a red stimulus with their left hand and a green stimulus with there right hand, then they are faster to respond and make fewer errors when the red stimulus appears on the left of the screen and the green stimulus appears on the right of the screen [46]–[47]. It has been argued that S-R compatibility effects may also influence imitation [47]. Subjects are faster to respond and make fewer errors when imitating actions when the hand performing the action is spatially compatible with the action being imitated. The S-R compatibility mechanism would argue that any modulations in the beta attenuation would only be modulated by the relative spatial location of the observed actions. This is in contradistinction to the goal-directed account [48]–[50]. According to the goal-directed model observed actions are covertly simulated by a motor representation reflecting the most easy or available response allowing the same goal. In relation to the current study this account would predict that subjects would covertly simulate a response with the left hand when the observed action occurred on the left of the screen and covertly simulate a response with the right hand when the observed action occurred on the right of the screen. However, the experimental design employed in the current study is unable to disambiguate between the S-R compatibility and the goal-directed account. However, the results of the current study are not compatible with the perspective taking account [51], [52]. According to the perspective taking account subjects covertly simulate a motor representation of the observed action with the same effector as the observed action as if they were performing the action. The perspective taking account would predict a significant effect main effect of the hand observed in **Experiment 2** and not the observed interaction between the hand observed and the direction the actor was facing.

### Summary

The attenuation of the *beta* oscillations during action observation has been interpreted as evidence of a MNS in humans. In agreement with previous studies we have shown that during the period when subjects were observing the movements there was a significant attenuation of *beta* oscillations overlying left and right sensorimotor cortices. In **experiment** 1 we found that *beta* oscillations overlying the left and right hemispheres were differentially attenuated by the hand that was observed moving with the attenuation of *beta* oscillations during action observation greatest overlying the sensorimotor cortex ipsilateral to the hand that was observed moving. This pattern of *beta* attenuation is the opposite of that previously reported during action execution [19], [22]. In **experiment 2** we found that this pattern of attenuation was driven by the side of the screen on which the observed movement occurred and not by the hand that was observed moving. *Beta* oscillations were more attenuated at sensors overlying the sensorimotor cortex contralateral to the side of the screen on which the observed movement occurred. These results are consistent with the firing of mirror neurons in area F5.

## Materials and Methods

We present data from two experiments. The analysis of both experiments was identical.

### Experiment 1

Data were recorded from 15 subjects (9 males, age range 25–45 yrs). All subjects gave written informed consent prior to testing and the recordings had local ethical committee approval (National Hospital for Neurology and Neurosurgery and Institute of Neurology NHS research ethics committee). Subjects sat in a dimly lit room and watched a series of short video clips (each lasting 5 s). In each video clip the subjects saw an actor making a movement with either their left or right hand from their side up to their ear [48]. The video clips showed one of five actors performing one of 28 different movements, in total there were 140 unique videos. In half the videos at the end of the movement the actor touched their ear and in the other half they did not. The subject's task was to judge whether the actor had touched their ear or not. In the current study, as in a previous study [45], there was no effect of the observed action goal on cortical oscillatory power so all videos were collapsed across this condition. This left 8 different classes of video that made up a 2×2×2 factorial design where the factors were: The hand the actor moved (right or left) the direction the head was facing (towards the camera or towards the corner of the room) and direction the eyes were looking (towards the camera or towards the corner of the room). For both the eyes away and the head away conditions actors looked away to the right and to the left. There were no significant effects for the direction the eyes were looking. Therefore all data presented here will collapse across this factor to leave a 2×2 factorial design (see Fig. 1A) where the factors were hand (left or right) and head direction (towards or away). An example of the experimental design is shown in Fig. 1A collapsed across the factors goal and eye direction. Each trial started with a blank screen with a fixation cross positioned centrally in the top half of the screen, in the position where the actors head would be (figure 1B). Subjects were instructed to fixate the cross and then fixate on the actor's head when the video began. After 1000 ms the video started. The first 1000 ms showed the actor hands by their sides with their eyes shut. After 1000 ms the actors opened their eyes. After a further 1000 ms subjects saw the actor move either their right or left hand. In all clips the actor moved their arm out sideways from their body and upwards so their hand ended near their ear. In all video clips the movement lasted exactly 2 s. In half the video clips the movement ended with the actor holding their ear and in the other half the movement ended with the actor's hand next to but not touching their ear. The last frame was held on the screen for 1 s. Subjects were instructed to watch the video clips, fixating on the actor's head throughout. At the end of each video clip the subjects were asked the following question on the screen, “Did the actor touch their body?” The subjects were instructed to answer by pressing a button with either their left or right index finger. After the question appeared, subjects were instructed as to which button response corresponded to ‘Yes’ and ‘No’. This prevented subjects from preparing the response movement during the period when they were watching the actor's movements.

### Recordings

MEG was recorded using 275 3^rd^ order axial gradiometers with the Omega275 CTF MEG system (VSMmedtech, Vancouver, Canada) at a sampling rate of 480 Hz. The video clips were projected onto a screen positioned approximately 1.5 m away for the subject. Maximal eccentricity of the videos was 4.5°. Eye-movements were recorded throughout to ensure the subjects maintained central fixation. Subjects performed five sessions consecutively. In each session subjects performed 64 trials, 16 trials for each of the 4 cells in the 2×2 design shown in Figure 1a. In total there were 80 trials for each of the conditions of interest.

All MEG analysis was performed in SPM5 (Wellcome Department of Imaging Neuroscience, London, UK. www.fil.ion.ucl.ac.uk/spm). First the data were epoched relative to the onset of the first frame of the video clip with the actors' eyes open. A time window of −2000 to 4000 ms was analysed (Fig. 1B). The data was band-pas filtered between 1–45 Hz and then downsampled to 100 Hz. Quantification of the oscillatory activity was performed using a wavelet decomposition of the MEG signal. The wavelet used was the complex Morlet's wavelet. The wavelet decomposition was performed across a 1–45 Hz frequency range. The wavelet decomposition was performed for each trial, for each sensor, and for each subject. These time-frequency maps were subsequently averaged across trials of the same task type thus producing a time-frequency map for each sensor for each condition. All statistical analysis was performed in sensor space. For the statistical parametric sensor space maps the time-frequency plots at each sensor, for each subject, were averaged across two frequency ranges; an *alpha* frequency range, defined here as 7–12 Hz, and a *beta* frequency range, defined here as 15–30 Hz. This analysis produced two time series of *alpha* and *beta* power respectively per sensor per condition per subject. For each frequency band, for each subject and for each trial type the time-series from each sensor was interpolated to produce a 2-D scalp map of the data at every time point. This interpolation assumed that all sensors were in the same place with respect to each subjects head. To ensure this assumption was valid care was taken to ensure that each subject was aligned in a similar manner with respect to the dewar when the data was collected. These 2D scalp maps at every time point were stacked to produce a 3D map where the third dimension was time [53] These maps were smoothed using a Gaussian kernel (FWHM 8×8 a.u. and 160 ms; [45]) prior to analysis at the second level. Contrasts of these images were taken at the second level with a design matrix including a subject specific regressor and correcting for heteroscedasticity across conditions. Contrasts were considered significant at p<0.005.

### Experiment 2

**Experiment 2** was a reanalysis of a previously published study [45]. Whereas the previous report focused on modulations in the *alpha* frequency band here we focused on the analysis of the *beta* frequency band. Detailed experimental details can be found in [45]. In brief, data were recorded from 14 subjects (9 males, age range 25–45 yrs). All subjects gave informed written consent prior testing and the recordings had local ethical committee approval. Subjects sat in a dimly lit room and watched a series of short video clips (each lasting 4 s). As in **experiment 1**, in each video clip the subjects saw an actor making a movement with either their left or right hand from their side up to their ear. The video clips showed one of five actors performing one of eight different movements. The eight movements made up a 2×2×2 factorial design where the factors were: The view of the actor (whether the actor was facing towards or away from the subject) the hand used (right or left) and the goal (whether at the end of the movement the actor touched their ear or not). The subject's task was to judge whether the actor had touched their ear or not. An example of the experimental design is shown in Figure 4A collapsed across the factor goal. All actors performed each of the eight movements. Each trial started with a blank screen with a fixation cross positioned centrally in the top half of the screen, in the position where the actors head would be (see Figure 4B). Subjects were instructed to fixate the cross and then fixate on the actor's head when the video began. After 500 ms the video started. In half the video clips the actor was facing towards the camera and in the other half was facing away. The first frame was played for 1000 ms before each clip was played. In half of the clips when the video was played the subjects saw the actor move their right hand and in the other half their left hand. In all clips the actor moved their arm out sideways from their body and upwards so their hand ended near their ear. In all video clips the movement lasted exactly 2000 ms. In half the video clips the movement ended with the actor holding their ear and in the other half the movement ended with the actor's hand next to but not touching their ear. The last frame was held on the screen for 1 s. Subjects were instructed to watch the video clips, fixating on the actor's head throughout. As in **experiment 1**, at the end of each video clip the subjects were asked the following question on the screen “Did the actor touch their body?” The subjects were instructed to answer by pressing a button with either their left or right index finger. After the question appeared the subjects were instructed as to which button response was for ‘Yes’ and which for ‘No’. The data was analysed in an identical manner to **experiment 1**.
